# Recurrent and transformation of borderline to malignant phyllodes tumour with osteoid differentiation: a case report and literature review

**DOI:** 10.3389/fonc.2024.1377074

**Published:** 2024-06-20

**Authors:** Navin Raj Balachandran, Norlia Abdullah, Muhammad Ishamuddin Ismail, Yin Ping Wong, Mohd Imree Azmi

**Affiliations:** ^1^ Department of Surgery, Medical Faculty, Universiti Kebangsaan Malaysia, Hospital Canselor Tuanku Muhriz, Kuala Lumpur, Malaysia; ^2^ Department of Pathology, Medical Faculty, Universiti Kebangsaan Malaysia, Hospital Canselor Tuanku Muhriz, Kuala Lumpur, Malaysia; ^3^ Department of Radiology, Medical Faculty, Universiti Kebangsaan Malaysia, Hospital Canselor Tuanku Muhriz, Kuala Lumpur, Malaysia

**Keywords:** recurrent, transformation, phyllodes, borderline, malignant, osteoid, differentiation

## Abstract

Phyllodes tumours or cystosarcoma phyllodes are fibroepithelial tumours of the breast and represent 1% of breast tumours. A 20-year-old nullipara presented with an enlarging left breast mass over 6 months. Although widely excised, it was reported to be a 12 × 10 × 5.5-cm borderline phyllodes tumour with involvement of the superior and inferior margins. Seven months later, she presented with a new ipsilateral breast lump measuring 8.5 × 7.5 × 4.6 cm. She underwent a left mastectomy, a three-rib resection with titanic rods for the thoracic cage reconstruction, and a latissimus dorsi flap wound closure. Histopathology revealed a high-grade malignant phyllodes tumour with features of osteoid differentiation with the nearest deep margin measuring 3 mm. She developed metastasis to the ipsilateral axillary lymph nodes and contralateral lung 2 months postoperatively. She was given palliative radiotherapy 60 Gy in 30 fractions to the left axilla. She developed sudden lower-limb weakness due to spinal metastases. The symptoms resolved with radiotherapy to the thoracic spine (T4–T8). As the lesion continued to grow rapidly from the anterior chest wall encircling towards the back, it was deemed unresectable. She was given palliative chemotherapy (doxorubicin six cycles, followed by ifosfamide one cycle) but had disease progression. She passed away 3 months later. The mainstay of treatment for phyllodes tumour is excision with a minimal margin of 1 cm. Although margins were involved after the first surgery, she was followed up as the pathology was a borderline phyllodes. When the lump recurred and had transformed, despite extensive surgery, it returned shortly and progressed. A borderline phyllodes should be excised to obtain a minimal margin of 1 cm, even if it means performing a mastectomy, to minimise recurrence. A recurrence may undergo malignant transformation which is largely chemotherapy and radiotherapy resistant. This will result in a poor outcome and decreased survival.

## Introduction

Phyllodes tumours, or cystosarcoma phyllodes, are fibroepithelial tumours of the breast. We report a case of a borderline phyllodes tumour with recurrence and transformation into a high-grade malignant phyllodes tumour.

## Case report

A 20-year-old Malay nullipara presented to the surgical clinic with a rapidly growing left breast mass over 6 months. She had no family history of any malignancies. Although the breast lump was widely excised, it was reported to be a 12 × 10 × 5.5-cm borderline phyllodes tumour with involvement of the superior and inferior margins. She was given the option of undergoing further surgery (re-excision of margins or even a mastectomy) or close follow-up. She opted for the latter. Seven months later, she presented with a new ipsilateral breast lump.

**Figure 1:**
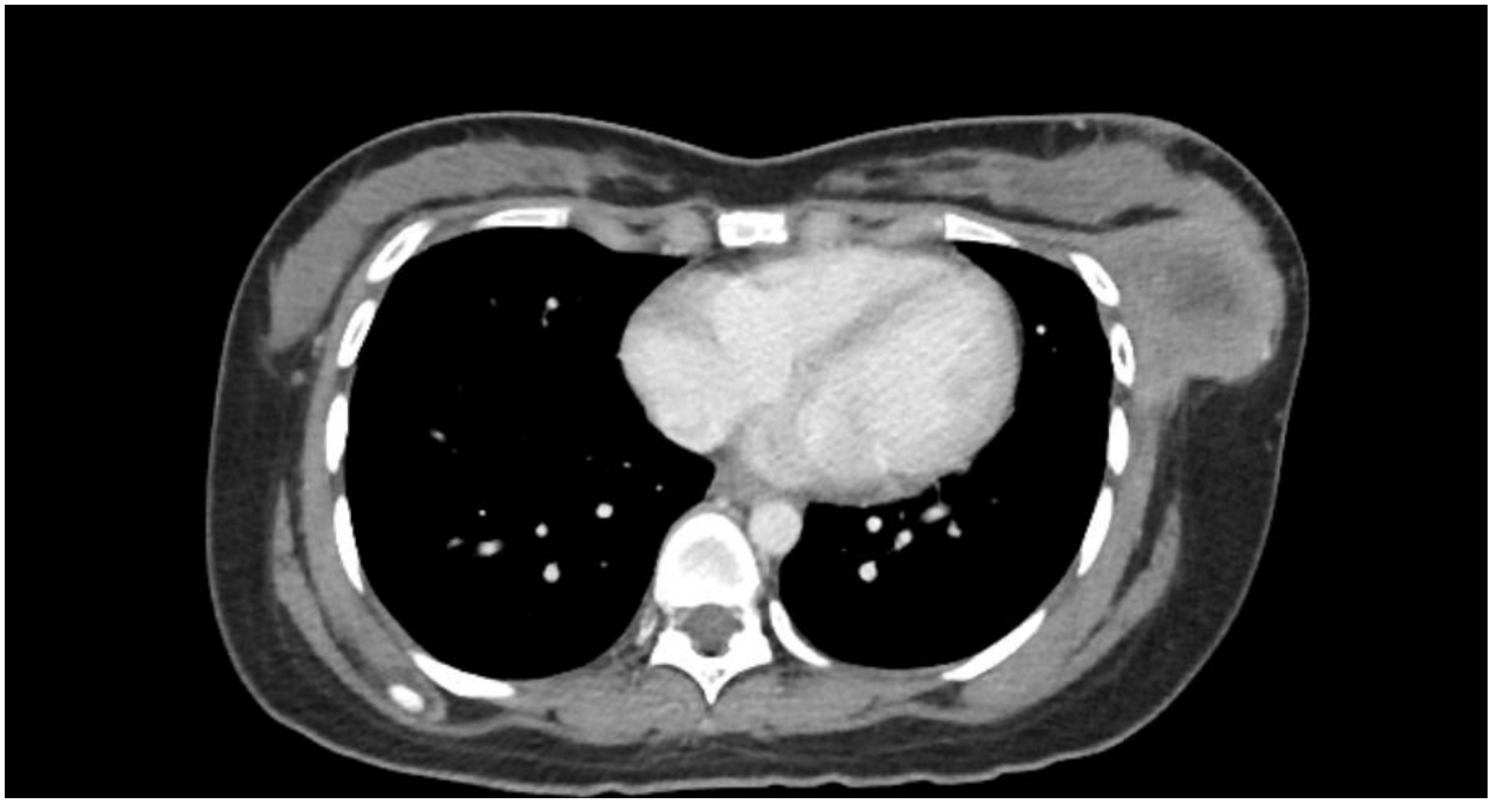
Computed tomography of the thorax showing a large tumour of the left breast.

**Figure 2:**
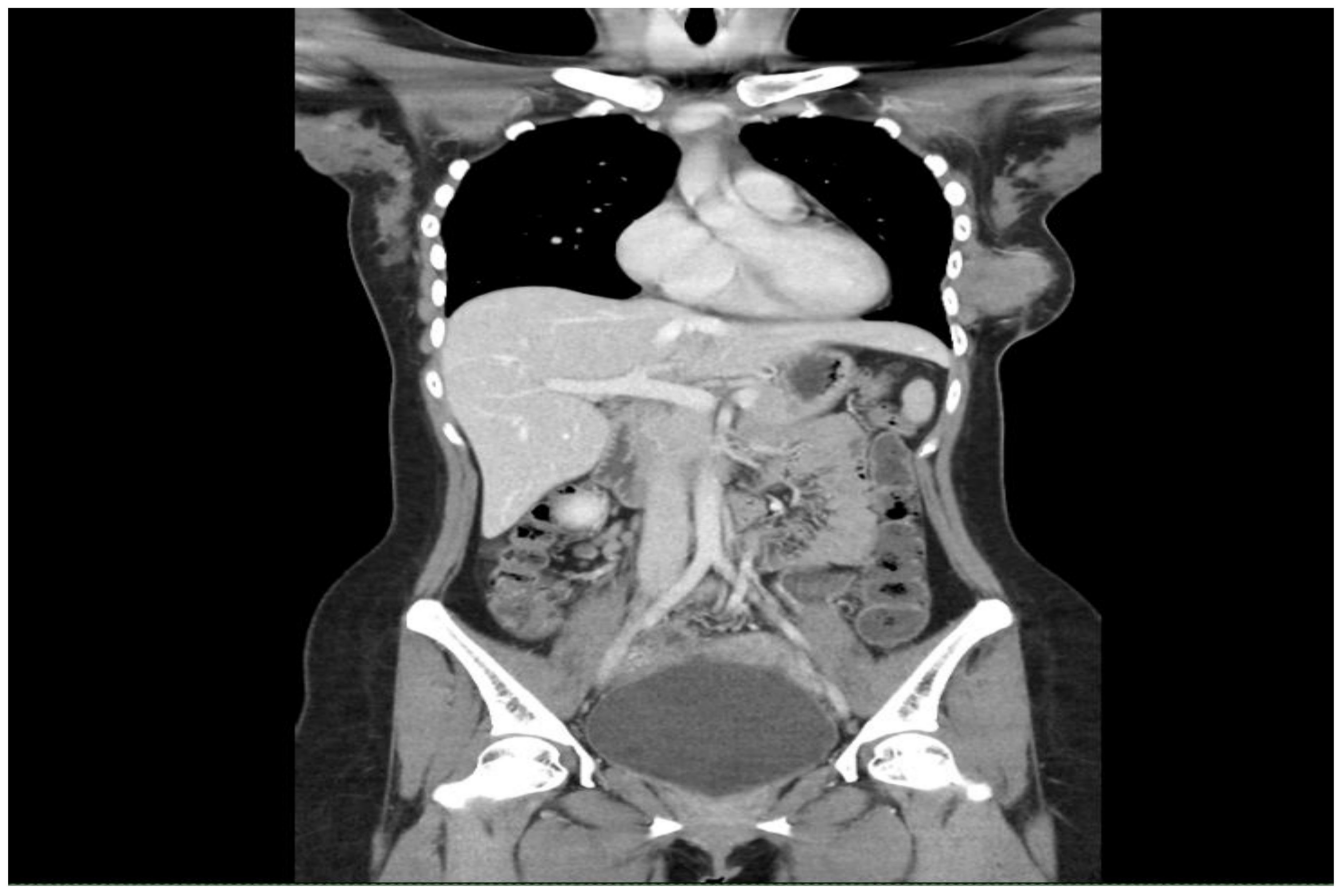
Computed tomography of the thorax, abdomen and pelvis showing the left breast lesion with extension into the pectoralis major, serratus anterior, ribs and pleura.

Examination revealed a firm, fixed mass measuring 7 × 6 cm, at the lower outer quadrant of the left breast with a well-healed hypertrophic scar from the previous surgery. There were no axillary or supraclavicular lymph nodes palpable. A CT of the thorax and abdomen revealed a large left breast tumour measuring 5.7 × 5.4 × 4.9 cm (**Figure 1**) with extension into the pectoralis major and serratus anterior muscles as well as the ribs and pleura (**Figure 2**), confirming recurrence. Biopsy of the mass confirmed it to be a malignant phyllodes tumour.

**Figure 3:**
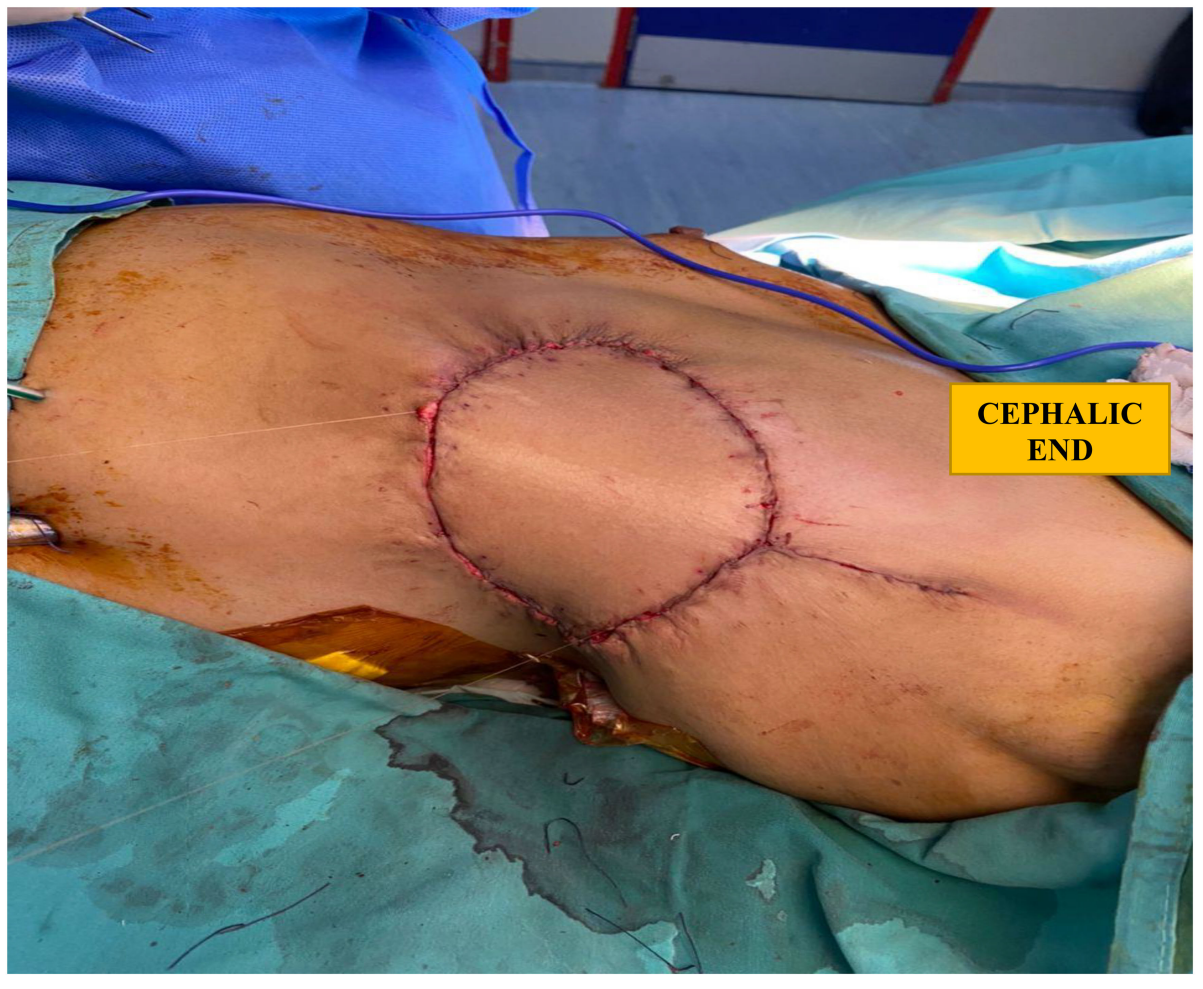
Closure of soft tissue defect, post reconstructed rib cage and mastectomy, with a latissimus dorsi flap.

She underwent a left mastectomy, a three-rib resection requiring several titanic rods for the thoracic cage reconstruction, and a latissimus dorsi flap for wound closure. The tumour which measured 15 × 15 cm was dissected en bloc with the lateral part of the pectoralis major, small part of the anterior and superior left serratus anterior, as well as the left anterolateral fifth to eighth ribs with intercostal muscles. Those ribs were resected with a 1-cm margin, and the pleural defect was repaired using a PROLENE mesh. Three contour plates of the MatrixRIB system were screwed to ribs sixth to eighth. The wound was closed using a latissimus dorsi (LD) flap (**Figure 3**). Axillary surgery was not performed as there were no enlarged nodes assessed clinically or via imaging (axillary ultrasound or CT scan). Phyllodes tumours are also known to not typically spread to the lymphatic system. She was subsequently discharged well on the eighth postoperative day. She was reviewed in our clinic a month postoperatively, and her surgical scars were well-healed.

Histopathology revealed a high-grade malignant phyllodes tumour with features of osteoid differentiation with the nearest margin (deep) measuring 3 mm. Microscopic assessment of the excised ribs revealed no evidence of malignancy. Results on the malignant cells showed to be negative for ER, and the proliferative index Ki-67 was high (50%). The tumour was not tested for HER2.

Two months postoperatively, she developed enlarged left axillary lymph nodes. An ultrasound measured the largest lymph node to be 1.34 × 1.85 cm (AP X W) with cortical thickness of 0.981 cm. An ultrasound-guided biopsy of that lymph node revealed high-grade malignant tumour, likely to be metastasis from the left breast malignant phyllodes tumour. A CT Thorax, Abdomen and Pelvis (TAP) revealed presence of suspicious right lung metastasis. She was given palliative radiotherapy 60 Gy in 30 fractions to the left axilla to prevent disease progression.

In the midst of undergoing radiotherapy, she developed sudden bilateral lower-limb weakness with reduction in sensation from dermatomal levels T4 to T8. This was as a result of spinal cord compression due to spinal metastasis. Her symptoms resolved with radiotherapy 20 Gy in five fractions to the thoracic spine (T4–T8). Several weeks later, the lesion continued to grow rapidly from the anterior chest wall encircling her trunk towards the back, making it unresectable.

CT TAP revealed worsening disease progression as evidenced by multiple new extra-thoracic (left supraclavicular, left anterior, and posterior chest walls), bilateral pulmonary, and rib metastasis. She was given palliative chemotherapy (doxorubicin six cycles, followed by ifosfamide one cycle), but she passed away 3 months later.

## Discussion

Phyllodes tumour (PT) of the breast is a unique entity and was first coined by Johannes Muller in 1838, using the term cystosarcoma phyllodes (1). PTs of the breast are rare and account for less than 1% of all primary breast tumours of the breast. Benign PTs can be difficult to distinguish from fibroadenomas. Phyllodes tumours are believed to arise from fibroadenomas or share a common precursor. Those with a history of fibroadenomas have an increased risk of developing phyllodes tumours (2). Radiation therapy can induce DNA damage and genetic alterations, predisposing individuals to develop phyllodes tumours. Our patient did not have any history of a prior breast lump or radiotherapy. Syndromes such as Li-Fraumeni syndrome, characterised by mutations in the TP53 gene, may increase the risk of developing phyllodes tumours. Such individuals often have a positive family history of breast cancer, which is not the case in our patient. Thus, she did not undergo any genetic tests.

Malignant PTs can grow in size quickly and metastasise early. Hence, clinical evaluation of these tumours is crucial and malignancy should be entertained specially in fast growing large lesions even if they look cytologically benign/borderline (3).

Phyllodes tumours are biphasic tumours, histologically characterised by a leaflike architecture resulting from an enhanced intracanalicular growth pattern, cleft-like spaces lined by epithelium and hypercellular stroma (4). The classification of PT as proposed by the World Health Organization (WHO) is divided into benign, borderline, and malignant. This is based on a combination of several histologic features, including stromal cellularity, nuclear atypia, mitotic activity, stromal overgrowth, and tumour margin appearance (5). It is very unusual to note heterologous osteoid differentiation in PTs. This is because heterologous differentiation such as chondrosarcoma, osteosarcoma, angiosarcoma, leiomyosarcoma, rhabdomyosarcoma, and liposarcoma are rarely encountered in malignant phyllodes tumours (6).

Classical phyllodes tumours typically have a biphasic pattern with stromal and epithelial components. Treatment usually involves surgical resection with wide margins. Adjuvant therapies such as radiation or chemotherapy may be considered for high-grade or recurrent tumours. Phyllodes tumours with osteoid heterologous differentiation are characterised by the presence of osteoid tissue within the tumour, indicating a more aggressive histological variant. While survival data specific to this subtype may be limited, the presence of osteoid differentiation suggests a more aggressive behaviour, potentially leading to poorer outcomes compared with classical phyllodes tumours (7)..

The majority of phyllodes tumours occur between the age of 35 and 45 years, and our patient was much younger than that stated in most literature (8). The percentage of malignant phyllodes tumour ranges from 23% to 50% and the prevalence of local recurrence is on the average 21%, with ranges of 10% to 17%, 14% to 25%, and 23% to 30% for benign, borderline malignant, and phyllodes tumours, respectively (9). Distant metastasis occurs in 10% of these tumours, and the commonest site are the lungs. Other less common organs involved are the bone, liver, and myocardium (10).

The mainstay of treatment for phyllodes tumour is excision with a minimal margin of 1 cm (11). Although margins were involved after the first surgery, our patient was closely followed up as it was a borderline phyllodes. When the lump grew and transformed, despite extensive surgery, it soon recurred and even metastasised to ipsilateral axillary lymph nodes. This is very rare, as unlike carcinomas, phyllodes tumours metastasise haematogenously and not by lymphatic spread (12).

In cases of high tumour-to-breast ratios or recurrent tumours, mastectomy is recommended (13). At the preoperative multidisciplinary team meeting involving the radiologist, breast surgeon, cardiothoracic surgeon, pathologist, and oncologist, it was agreed that the CT scan revealed a tumour with extension into the pectoralis major, serratus anterior muscles, and the ribs and pleura. We decided to proceed with a left mastectomy and en bloc resection of the lateral part of the pectoralis major and the small part of the anterior and superior serratus anterior, and rib resection followed by rib reconstruction and wound closure using an LD flap.

Several options exist for reconstruction of the anterior chest wall defect. With a large defect such as in our case (14–16), a muscle flap was preferable. Muscle flap options commonly used in breast surgery are LD and rectus abdominis (17). For our patient, the pedicled LD flap provided an ideal solution as it required minimal surgical time and no microvascular anastomosis was required with its associated risks. As the bony defect exceeded three ribs (greater than 5cm), a stable structural support was required and there were no randomised control trials (18) obtained using the MatrixRIB system. The main goals of reconstruction are prevention of a flail chest, maintenance of physiologic respiration, protection of thoracic organs, and an acceptable cosmetic result (18).

Adjuvant radiotherapy reduces the local recurrence rates of borderline and malignant phyllodes tumours but does not affect overall or disease-free survival (19). The role of adjuvant chemotherapy is uncertain mainly due to the fact that there are no randomised control trials to support it. In comparison, malignant phyllodes tumours have a better prognosis than most high-grade sarcomas of a similar stage (20).

Studies have identified several molecular alterations in phyllodes tumours. MED12 mutations are common and are thought to play a role in tumour initiation. TERT promoter mutations are associated with poor prognosis and may promote tumour progression and metastasis. p53 mutations are involved in tumorigenesis and may confer resistance to certain treatments (21, 22).

## Conclusion

Borderline phyllodes tumours should be excised to obtain a minimal margin of 1 cm, even a mastectomy, to minimise recurrence. Phyllodes tumours are largely chemotherapy and radiotherapy resistant. The prognosis for malignant phyllodes is adverse, with medial overall survival ranging from 5 to 17 months (23).

## Limitations of this article

Molecular studies have not been performed on these tumour cells.

## Data availability statement

The original contributions presented in the study are included in the article/supplementary material. Further inquiries can be directed to the corresponding author.

## Ethics statement

Written informed consent was obtained from the individual(s) for the publication of any potentially identifiable images or data included in this article.

## Author contributions

NB: Writing – original draft, Conceptualization. NA: Writing – review & editing, Supervision, Project administration, Funding acquisition. MII: Writing – review & editing. WYP: Writing – review & editing. MA: Writing – review & editing.
